# *Clerodendrum volubile* Ethanol Leaf Extract: A Potential Antidote to Doxorubicin-Induced Cardiotoxicity in Rats

**DOI:** 10.1155/2020/8859716

**Published:** 2020-07-04

**Authors:** Olufunke Esan Olorundare, Adejuwon Adewale Adeneye, Akinyele Olubiyi Akinsola, Daniel Ayodele Sanni, Mamoru Koketsu, Hasan Mukhtar

**Affiliations:** ^1^Department of Pharmacology and Therapeutics, Faculty of Basic Medical Sciences, College of Health Sciences, University of Ilorin, Ilorin, Kwara, Nigeria; ^2^Department of Pharmacology, Therapeutics and Toxicology, Faculty of Basic Clinical Sciences, Lagos State University College of Medicine, 1-5 Oba Akinjobi Way, G.R.A., Ikeja, Lagos, Nigeria; ^3^Department of Pathology and Forensic Medicine, Faculty of Basic Clinical Sciences, Lagos State University College of Medicine, 1-5 Oba Akinjobi Way, G.R.A., Ikeja, Lagos, Nigeria; ^4^Department of Chemistry and Biomolecular Science, Faculty of Engineering, Gifu University, 1-1 Yanagido, Gifu 501-1193, Japan; ^5^Department of Dermatology, University of Wisconsin-Madison, Medical Science Center, 1300 University Avenue, Madison, WI 53706, USA

## Abstract

Doxorubicin is widely applied in hematological and solid tumor treatment but limited by its off-target cardiotoxicity. Thus, cardioprotective potential and mechanism(s) of *CVE* in DOX-induced cardiotoxicity were investigated using cardiac and oxidative stress markers and histopathological endpoints. 50–400 mg/kg/day *CVE* in 5% DMSO in distilled water were investigated in Wistar rats intraperitoneally injected with 2.5 mg/kg DOX on alternate days for 14 days, using serum troponin I and LDH, complete lipid profile, cardiac tissue oxidative stress marker assays, and histopathological examination of DOX-treated cardiac tissue. Preliminary qualitative and quantitative assays of *CVE*'s secondary metabolites were also conducted. Phytochemical analyses revealed the presence of flavonoids (34.79 ± 0.37 mg/100 mg dry extract), alkaloids (36.73 ± 0.27 mg/100 mg dry extract), reducing sugars (07.78 ± 0.09 mg/100 mg dry extract), and cardiac glycosides (24.55 ± 0.12 mg/100 mg dry extract). 50–400 mg/kg/day *CVE* significantly attenuated increases in the serum LDH and troponin I levels. Similarly, the *CVE* dose unrelatedly decreased serum TG and VLDL-c levels without significant alterations in the serum TC, HDL-c, and LDL-c levels. Also, *CVE* profoundly attenuated alterations in the cardiac tissue oxidative stress markers' activities while improving DOX-associated cardiac histological lesions that were possibly mediated via free radical scavenging and/or antioxidant mechanisms. Overall, *CVE* may play a significant therapeutic role in the management of DOX-induced cardiotoxicity in humans.

## 1. Introduction

Drug-induced cardiotoxicity remains the most widely studied of drug-induced organ toxicities [1]. Several approved drugs and agrochemicals have been implicated in these toxicities, including acetaminophen, gentamicin, rifampicin, carbon tetrachloride, and antineoplastic drugs [2]. Thus, accidental, acute overdose, or chronic use of some of these drugs could result in multiorgan toxicities including hepatotoxicity, nephrotoxicity, cardiotoxicity, and testicular/ovarian toxicity. These specific organs are known to be mostly susceptible and are organs of metabolism and excretion of these drugs [3]. Despite extensive studies of these organ toxicities, definitive therapeutic/prophylactic options at ameliorating the deleterious effects of these drugs are still limited. In view of this, research into the etiopathology and development of alternative or complementary options from traditional medicine is currently been encouraged [4]. Also, the World Health Organization reported that over 80% of the world population depends partly or wholly on medicinal plant-derived pharmaceuticals [5].

Doxorubicin (DOX) is a broad-spectrum antibiotic anthracycline with wide application in the clinical chemotherapeutic management of solid tumors such as breast, lung, ovarian, and uterine cancers and leukemias [6]. Despite its incontrovertible efficacy, DOX is notorious for its life-threatening toxicity profile such as neurotoxicity, hepatotoxicity, hematotoxicity, and cardiotoxicity, thus limiting its clinical use in cancer treatment. Life-threatening cardiomyopathy represents the cumulative dose-limiting toxicity of the drug. DOX toxicities are majorly attributed to the interplay of oxidative stress and free radical formation from its highly reactive and toxic secondary metabolites [7].

In African traditional medicine, several medicinal plants are reputed for effectively ameliorating the deleterious effects of drug poisoning on organs such as the liver and the kidneys. These medicinal plants (in whole or parts) include *Phyllanthus amarus*, *Harungana madagascariensis*, *Carica papaya* leaves, *Zingiber officinale* rhizome, *Vernonia amygdalina* leaves, and *Garcinia kola* seeds [8, 9]. However, the folkloric therapeutic claims of some of these plants have been scientifically verified and reported. For example, the protective activities of various plant extracts of *Phyllanthus amarus* [10–12], *Harungana madagascariensis* [13], *Carica papaya* leaves [14, 15], *Zingiber officinale* rhizome [16–19], *Vernonia amygdalina* leaves [20, 21], and *Garcinia kola* seeds [22, 23] against drug-induced hepato- and nephrotoxicities are well documented. Similarly, several studies have equally reported ameliorative effects of medicinal plants against drug-induced cardiotoxicity [24–28].

*Clerodendrum volubile* P. Beauv, popularly known as white butterfly, belongs to the Lamiaceae family. In Nigeria, the plant is locally known as “Marugbo” or “Eweta” among the Ikale, Ilaje, and Apoi people of Ondo State (Southwest Nigeria) and “Obnettete” among the Itsekiri and Urhobo tribes (Niger Delta area of Nigeria) where it is often combined with other vegetables as a condiment and spice to improve taste and aroma [29]. *Clerodendrum volubile* is indigenous and ubiquitous to the riverine belts of the West African tropical rainforest of Nigeria, Benin Republic, Ghana, Cameroon, Burkina Faso, and Sierra Leone where it is also grown as an ornamental plant [30, 31]. Due to its high nutritive and ethnopharmacological value, *Clerodendrum volubile* whole plant and its parts are used in the African ethnomedicine in the local management of joint pains and swellings, diabetes mellitus, gastric ulcer, obesity and hyperlipidemia, hypertension, and other heart diseases and dropsy [31, 32]. However, some of these folkloric claims have been scientifically validated and have been attributed to its high polyphenol content such as ajugoside, pectolinarigenin, protocatechuic acid, biochanin, and 5, 7, 4′-trimethoxykaempferol [31]. *Clerodendrum volubile* has also been reported to exhibit potential role in the management of human breast cancer by inhibiting cell cycle phases, especially the *G*_0_/*G*_1_ phase, cell proliferation, and decreasing the expression of matrix metalloproteinase 9 enzymes [33], as well as the ability of its extracts to scavenge free radicals, especially reactive oxygen species, generated by human breast cancer cell lines *in vitro* [33] and its antiproliferative activity against prostate cancer cells [34]. Considering the wide application and historical use of different plant parts of *Clerodendrum volubile* in the traditional management of heart diseases, the reported role of reactive oxidative stress in the etiopathogenesis of doxorubicin-related heart disease, and paucity of scientific validation of the plant use in the management of drug-related heart diseases including drug-induced cardiotoxicity, the present exploratory study is, therefore, designed at evaluating the possible antidotal potential of 100–400 mg/kg of the *Clerodendrum volubile* ethanol leaf extract (*CVE*) in doxorubicin- (DOX-) induced cardiotoxic rats for 14 days. In doing this, reliable cardiac injury biomarkers (serum troponin I and LDH), indices of cardiovascular disease, as well as histopathological studies of *CVE*-DOX-treated cardiac muscles, were evaluated. In addition, the effect of *CVE* on SOD, CAT, GSH, GPx, GST, and MAD activities in the DOX-treated cardiac tissues was evaluated.

## 2. Materials and Methods

### 2.1. Plant Materials

Stock of fresh mature whole plants of *Clerodendrum volubile* was purchased from Herbal Vendors in the Isikan Market in Akure, Ondo State, Nigeria, in the month of March 2019. Samples of the plant obtained were subjected to botanical identification and referencing at the University of Ilorin (UNILORIN) Herbarium, with the voucher specimen number UILH/01/019/1254 allotted.

### 2.2. Extraction Process

Fresh leaves of *Clerodendrum volubile* were destalked from the whole plant and then gently but thoroughly rinsed under running tap water and completely air-dried at the room temperature (28–33°C) until the weight of the dried leaves was constant. The dried leaves were then pulverized using the milling machine and kept in a water- and air-tight container.

1.50 kg of the pulverized leaves was completely macerated in 8 liters of absolute ethanol at room temperature for 5 days but intermittently shaken to ensure complete dissolution. Thereafter, the solution was first filtered with cotton wool and then with the 110 mm Whatman filter paper. The resultant filtrate was then concentrated *in vacuo* using a rotary evaporator (BUCHI Rotavapor® Model R-215, Switzerland) with the Vacuum Module V-801 EasyVac®, Switzerland) set at a revolution of 70 rpm and a temperature at 36°C before it was completely dried over a waterbath preset at 40°C. The jelly-like, dark-colored residue left behind was weighed and stored in an air- and water-proof container which was kept in a refrigerator at 4°C. From this stock, fresh solutions were made whenever required.

(1) $$\begin{array}{c} \%\text{ yield }=\frac{\text{weight of the crude extract obtained}\left(\text{g}\right)}{\text{weight of the starting pulverized dry leaf extracted }\left(\text{g}\right)}\times 100. \end{array}$$

### 2.3. Preliminary Qualitative Phytochemical Analysis

The presence of saponins, tannins, alkaloids, flavonoids, anthraquinones, glycosides, and reducing sugars was detected by the simple and standard qualitative and quantitative methods described by Trease and Evans [35] and Sofowora [36].

### 2.4. Quantitative Determination of Secondary Metabolites in *CVE*

#### 2.4.1. Preparation of Fat-Free Sample

2 g of *CVE* was exhaustively defatted with 100 ml of diethyl ether using the method earlier described by Edeoga et al. [37].

#### 2.4.2. Total Phenol Quantification

The total phenolic content in *CVE* was spectrophotometrically determined using the procedure previously described by Edeoga et al. [37].

#### 2.4.3. Alkaloid Quantification

The alkaloid content of *CVE* was determined using the method earlier described by Harborne [38]. 5 g of *CVE* was weighed into a 250 ml beaker, and 200 ml of 10% acetic acid in ethanol was added. The resulting solution was covered and allowed to stand for 4 hours, filtered, and then the filtrate was concentrated on a waterbath to one-quarter of the original volume. Concentrated ammonium hydroxide was added drop-wise to the concentrate until the precipitation was complete. The whole solution was allowed to settle, and the precipitate was collected and rinsed with dilute ammonium hydroxide and then filtered. The residue is the alkaloid, which was dried and weighed.

#### 2.4.4. Tannin Quantification

Tannin content of *CVE* was estimated using the method by Van-Burden and Robinson [39]. 500 mg of defatted *CVE* was weighed into a 50 ml plastic bottle. 50 ml of distilled water was added and shaken continuously for 1 hour on a mechanical shaker. This was filtered into a 50 ml volumetric flask and made up to the mark. Following this, 5 ml of the filtrate was pipetted out into a test tube and mixed with 2 ml of 0.1 M FeCl_3_ in 0.1 N HCl and 0.008 M potassium ferrocyanide. The absorbance of the resulting mixture was measured at 120 nm within 10 min.

#### 2.4.5. Saponin Quantification

Saponin was estimated by the method previously used by Obadoni and Ochuko [40]. 2 g of *CVE* was placed into a conical flask, and 10 ml of 20% aqueous ethanol was added. The resulting mixture was heated over a hot waterbath for 4 hours under continuous stirring at 55°C. The mixture was filtered, and the residue was re-extracted with another 20 ml of 20% ethanol. The combined filtrate was reduced to 4 ml over a waterbath at 90°C. The concentrate was transferred into a 50 ml separating funnel, and 2 ml of diethyl ether was added and shaken vigorously. The aqueous layer was recovered, while the ether layer was discarded. The extraction process was repeated one more time, and n-butanol was added to the combined aqueous portion. The resulting mixture was shaken and washed twice with 1 ml of 5% aqueous sodium chloride and filtered, while the resulting solution was heated over a waterbath. After evaporation, the samples were dried in the oven to a constant weight, and the saponin content was calculated as the percentage of the extract.

#### 2.4.6. Reducing Sugar Quantification

Reducing sugar content in *CVE* was determined using the spectrophotometric method as described by Shaffer and Somogyi [41].

#### 2.4.7. Quantitative Determination of Total Flavonoid Content in *CVE*

Total flavonoids in *CVE* were estimated using the method by Ordonez et al. [42]. To 1 ml of crude *CVE*, equivalent 1 ml of 2% aluminum chloride in the ethanol solution was added. After 1 hour of incubation at room temperature (28°C) for color development, the absorbance was measured at 420 nm using the ®Unico 2100 spectrophotometer (United Products and Instruments Inc., Shanghai, China). A golden yellow color indicated the presence of flavonoids. Total flavonoid contents were calculated as the rutin hydrate (minimum 98%; Sigma Chemicals Co., St. Louis, MO, USA) equivalent using the mathematical equation described by Ordonez et al. [42].

#### 2.4.8. Determination of Total Proanthocyanidin Contents in *CVE*

Total proanthocyanidin (tannin) content in *CVE* was estimated by the method of Sun et al. [43]. 0.5 ml of 50 mg/l of the extract was mixed in 3 ml of 4% vanillin-methanol solution and 1.5 ml of concentrated hydrochloric acid, and the mixture was allowed to stand for 15 minutes at room temperature (28°C) for color development. The absorbance was measured at 500 nm using the ®Unico 2100 spectrophotometer (United Products and Instruments Inc., Shanghai, China). Total proanthocyanidin contents were calculated as the catechin hydrate (minimum 98%) (Sigma Chemicals Co., St. Louis, MO, USA) equivalent (mg/g) using the mathematical equation described by Sun et al. [43].

#### 2.4.9. Determination of Total Phenols in *CVE*

Total phenol content in *CVE* was determined by the modified Folin–Ciocalteu method of Wolfe et al. [44]. An aliquot of each of *CVE* was mixed with 2.5 ml of the Folin–Ciocalteu reagent (previously diluted with distilled water, 1 : 10 v/v) and 2 ml of (75 g/l) of sodium carbonate. The tubes were vortexed for 15 seconds and allowed to stand for 30 minutes at 40°C for color development. Absorbance was measured at 765 nm using the ®Unico 2100 spectrophotometer (United Products and Instruments Inc., Shanghai, China). This procedure was replicated thrice. Total phenolic content was calculated and expressed as the mg/g rutin equivalent as earlier described by Wolfe et al. [44].

### 2.5. *In Vitro* Antioxidant Profiling of *CVE*

#### 2.5.1. Determination of DPPH-Scavenging Activity of *CVE*

The effect of *CVE* was estimated using the method by Liyana-Pathirana and Shahidi [45]. A solution of 0.135 mM 1, 1-diphenyl-2-picrylhydrazyl (DPPH) (Sigma Aldrich, St. Louis, USA) in methanol was prepared, and 1.0 ml of this solution was mixed with 1.0 ml of methanol containing 0.2–1.0 mg of each extract. The reaction mixture was vortexed thoroughly and left in the dark at room temperature for 30 min. The spectrophotometric absorbance of the mixture was measured at 517 nm. The reference drug, vitamin C (Sigma Chemicals Co., St. Louis, USA), used was equally prepared at the same concentration, and the experiment was conducted in triplicate. The ability to scavenge the DPPH radical was calculated by the following equation:

(2) $$\begin{array}{c} \text{DPPH radical}-\text{scavenging activity }\left(\%\right)=\frac{\left[\left({\text{AbS}}_{\text{control}}-{\text{AbS}}_{\text{sample}}\right)\right]}{{\text{AbS}}_{\text{control}}}\times 100, \end{array}$$

where Abs_control_ = absorbance of DPPH radical + methanol and Abs_sample_ = absorbance of DPPH radical + sample extract/standard.

#### 2.5.2. Determination of Superoxide Anion and Nitric Oxide-Scavenging Activities of *CVE*

Superoxide anion and nitric oxide-scavenging activities of *CVE* were evaluated using the method by Sreejayan and Rao [46]. In both assaying methods, quercetin was used as the standard drug.

### 2.6. Spectral Studies of Secondary Metabolites in *CVE* Using Gas Chromatography-Mass Spectrometry

GC-MS analysis was performed using a 7820A gas chromatograph coupled to a 5975C inert mass spectrometer (with triple axis detector) and an electron-impact source (Agilent Technologies, Santa Clara, CA 95051, USA). 0.5 g of *CVE* was suspended in ethanol to make a concentration of 100 mg/ml (w/v), followed by filtration through Varian Bond Elute C18 solid-phase extraction to remove impurities. The stationary phase of separation of the compounds was carried out on a HP‐5 capillary column coated with 5% of phenyl methyl siloxane (30 m length × 0.32 mm diameter × 0.25 *μ*m film thickness) (Agilent Technologies, Santa Clara, CA 95051, USA). The carrier gas used was GC-grade helium (99.999% purity) at a constant flow rate of 1.573 ml/min, an initial nominal pressure of 1.9514 psi, and at an average velocity of 46 cm/s. One microliter (1 *μ*l) of the samples was injected in the split-less mode at an injection temperature of 260°C. Purge flow was 21.5 ml/min at 0.50 min with a total gas flow rate of 23.355 ml/min; the gas saver mode was switched on. The oven was initially programmed at 60°C (1 min) and then ramped at 4°C/min to 110°C (3 min), followed by temperature program rates of 8°C/min to 260°C (5 min) and 10°C/min to 300°C (12 min). Run time was 56.25 min with a 3 min solvent delay. The mass spectrometer was operated in the electron‐impact ionization mode at 70 eV with an ion source temperature of 230°C, quadrupole temperature of 150°C, and transfer line temperature of 280°C. The mass spectrophotometer conditions are solvent delay of 3.00 min, gain factor of 1.00, and resulting EM voltage of 1859, and scanning of possible compounds was from m/z 30 to 550 amu at a 2.62 s/scan rate. Using computer searches on a National Institute Standard and Technology (NIST) 14 Mass Spectral Database and the Mass Spectral Search Program (Version 2.2) and comparing, the spectrum obtained through GC-MS compounds present in the *CVE* was identified. The spectrum of the unknown components was compared with the spectrum of known components stored in the NIST library to ascertain its chemical identity [47, 48].

### 2.7. Experimental Animals

Young adult male Wistar albino rats (aged 8–12 weeks and body weight: 130–190 g) used in this study were obtained from the Animal House of the Lagos State University College of Medicine, Ikeja, Lagos State, Nigeria, after an ethical approval (Protocol Identification Code: UERC/BMS/134 and UERC Approval Number: UERC/ASN/2019/1703) was obtained from the University of Ilorin Ethical Review Committee for Postgraduate Research. The rats were handled in accordance with international principles guiding the Use and Handling of Experimental Animals [49]. The rats were maintained on standard rat feed (Ladokun Feeds, Ibadan, Oyo State, Nigeria) and potable water which were made available *ad libitum*. The rats were maintained at an ambient temperature between 28 and 30°C, humidity of 55 ± 5%, and standard (natural) photoperiod of approximately 12/12 hours of alternating light and dark periodicity.

### 2.8. Measurement of Body Weight

The body weights of rats were taken on days 1 and 14 of the experiment and determined on a digital rodent weighing scale (®Virgo Electronic Compact Scale, New Delhi, India). The obtained values were expressed in grams (g).

### 2.9. Induction of DOX-Induced Cardiotoxicity and Treatment of Rats

Prior to commencement of the experiment, rats were randomly allotted into 8 groups of 7 rats per group such that the weight difference between and within groups was not more than ±20% of the average weight of the sample population of rats used for the study. However, the choice of the therapeutic dose range of 100, 200, and 400 mg/kg/day of *CVE* was made based on the result of the preliminary studies conducted.

In this experimental repeated dose model which lasted for 14 days, Groups I rats which served as the untreated control were orally pretreated with 10 ml/kg/day of distilled water but equally treated with 2.5 mg/kg of doxorubicin hydrochloride (®Celondoxily Injection 50, CELON Laboratories PVT. Limited, Gajularamaram, Ranga Reddy District-500 090, Telangana State, India) dissolved in 0.9% normal saline administered on alternate days for 14 days. Group II rats were orally treated with 200 mg/kg/day of *CVE* dissolved in 5% DMSO distilled water (*CVE* being only partly soluble in water, DMSO an organosulfur polar aprotic and inert solvent that readily dissolves both polar and nonpolar compounds was used) but treated with 1 ml/kg of 0.9% normal saline administered intraperitoneally on alternate days for 14 days. Group III–Group VI rats were orally pretreated with 50 mg/kg/day, 100 mg/kg/day, 200 mg/kg/day, and 400 mg/kg/day of *CVE* dissolved in 5% DMSO distilled water 3 hours before treatment with 2.5 mg/kg of doxorubicin in 0.9% normal saline administered intraperitoneally on alternate days for 14 days, respectively. Group VII rats which served as the positive control group were equally pretreated with 20 mg/kg/day of Vitamin C 3 hours before treatment with 2.5 mg/kg of doxorubicin in 0.9% normal saline administered intraperitoneally on alternate days for 14 days. Group VIII rats were the untreated normal rats and were orally pretreated with 10 ml/kg/day of distilled water 3 hours before treatment with 2.5 mg/kg of doxorubicin in 0.9% normal saline administered intraperitoneally on alternate days for 14 days (Table 1) [50, 51]. The choice of vitamin C was made because it is a standard antioxidant agent, and its effect as a positive control was compared with other treatment groups.

### 2.10. Blood Sample Collection

On the 14^th^ day which was the last day of the experiment, the rats were weighed and later fasted overnight but drinking water was made available *ad libitum*. Rats were sacrificed, and whole blood samples were collected directly from the heart under inhaled diethyl ether anesthesia. Blood samples were carefully collected with the fine 21G Needle and 5 ml Syringe (Hangzhou Longde Medical Products Co. Ltd., Hangzhou, China) without causing damage to the heart tissues. The rats' heart, liver, kidneys, and testes were identified, harvested, and weighed.

### 2.11. Biochemical Assays

Blood samples obtained directly from the heart chamber were allowed to clot and then centrifuged at 5000 rpm to separate clear sera from the clotted blood samples. The clear samples were obtained for assays of the following biochemical parameters: serum cardiac troponin I, LDH, TG, TC, and cholesterol fractions (HDL-c, LDL-c, and VLDL-c). Serum lipids were assayed using methods by Tietz [52], while serum troponin I and LDH were estimated by standard bioassay procedures.

### 2.12. Calculation of AI and CRI

AI was calculated as LDL-c (mg/dl) ÷ HDL-c (mg/dl) [53], while CRI was calculated as TC (mg/dl) ÷ HDL-c (mg/dl) [54].

### 2.13. Determination of Antioxidant Activities in the Rat Cardiac Tissues

After the rats were sacrificed humanely under inhaled diethyl ether, the heart was harvested *en bloc*. The heart was gently and carefully divided into two halves (each consisting of the atrium and ventricle) using a new surgical blade. The left half of the heart was briskly rinsed in ice cold 1.15% KCl solution in order to preserve the oxidative enzyme activities of the heart before being placed in a clean sample bottle which itself was in an ice-pack-filled cooler. This is to prevent the breakdown of the oxidative stress enzymes in these organs.

#### 2.13.1. Determination of SOD Activities in the Heart Tissues

Superoxide dismutase activity was determined by its ability to inhibit the auto-oxidation of epinephrine by the increase in absorbance at 480 nm as described by Paoletti et al. [55]. Enzyme activity was calculated by measuring the change in absorbance at 480 nm for 5 minutes.

#### 2.13.2. Determination of CAT Activities in the Heart Tissues

Tissue CAT activities were determined by the method described by Hadwan [56]. The specific activity of CAT was expressed as U/ml.

#### 2.13.3. Determination of GSH, GPx, and GST Activities in the Heart Tissues

The reduced glutathione (GSH) content in the heart tissue was estimated according to the method described by Rahman et al. [57]. To the homogenate, 10% TCA was added and centrifuged. One millilitre of the supernatant was treated with 0.5 ml of Elman's reagent (19.8 mg of 5, 5-dithiobisnitro benzoic acid (DTNB) in 100 ml of 0.1% sodium nitrate) and 3.0 ml of phosphate buffer (0.2 M, pH 8.0). The absorbance was read at 412 nm. Similarly, GPx and GST activities were determined using the method by Faraji et al. [58] and Vontas et al. [59].

#### 2.13.4. Determination of MDA Activities in the Heart Tissues

Method by Buege and Aust [60] was adopted in determining MDA activities in the cardiac tissue. One millilitre of the supernatant was added to 2 ml of (1 : 1 : 1 ratio) the TCA-TBA-HCl reagent (thiobarbituric acid 0.37%, 0.24 N HCl, and 15% TCA) boiled at 100°C for 15 minutes and allowed to cool. Flocculent material was removed by centrifuging at 3000 rpm for ten minutes. The supernatant was removed, and the absorbance was read at 532 nm against a blank. MDA was calculated using the molar extinction for the MDATBA-complex of 1.56 × 10^5^ m^−1^·cm^−1^.

#### 2.13.5. Histopathological Studies of the Heart

Using the remaining equally divided harvested heart, the right halves of the six randomly selected rats from each treatment and control groups were subjected to histopathological examinations, with the right ventricle being the most susceptible to doxorubicin toxicity of the heart chambers. After rinsing in normal saline, the dissected right half was preserved in 10% formo-saline before it was completely dehydrated in absolute (100%) ethanol. It was then embedded in routine paraffin blocks. From the embedded paraffin blocks, 4-5 *μ*m thick sections of the tissue were prepared and stained with the hematoxylin-eosin stain. These were examined under a photomicroscope (Model N-400 ME, CEL-TECH Diagnostics, Hamburg, Germany) connected with a host computer. Sections were illuminated with white light from a 12 V halogen lamp (100 W) after filtering with a 520 nm monochromatic filter. The slides were examined for associated histopathological lesions [61].

### 2.14. Statistical Analysis

Data were presented as mean ± SEM of four and seven observations for the *in vitro* and *in vivo* studies, respectively. Statistical analysis was done using two-way analysis of variance, followed by a *post hoc* test, Student–Newman–Keuls test, on GraphPad Prism Version 5. Statistical significance was considered at *p* < 0.05, *p* < 0.01, and *p* < 0.001.

## 3. Results

### 3.1. %yield

Complete extraction of the pulverized dry leaf sample of *Clerodendrum volubile* in absolute ethanol was calculated to be 8.39%. The resultant residue was a dark color, sticky, jelly-like, and sweet-smelling (bland) residue which was not completely soluble in water but completely soluble in methanol and ethanol.

### 3.2. Qualitative Phytochemical Analysis of *CVE*

Phytochemical analysis of *CVE* showed the presence of flavonoids, alkaloids, reducing sugars, and cardiac glycosides, while saponins, tannins, phenols, phlobatannins, steroids, and terpenoids were absent.

### 3.3. Quantitative Analysis of *CVE*

Table 2 shows the estimates of each of the secondary metabolites present in 100 mg of *CVE*. As indicated in Table 2, *CVE* contains flavonoids (34.79 ± 0.37 mg/100 mg of the dry extract), alkaloids (36.73 ± 0.27 mg/100 mg of the dry extract), reducing sugars (07.78 ± 0.09 mg/100 mg of the dry extract), and cardiac glycosides (24.55 ± 0.12 mg/100 mg of the dry extract).

### 3.4. Spectral Studies of Secondary Metabolites in *CVE* Using Gas Chromatography-Mass Spectrometry

**Figure 1:**
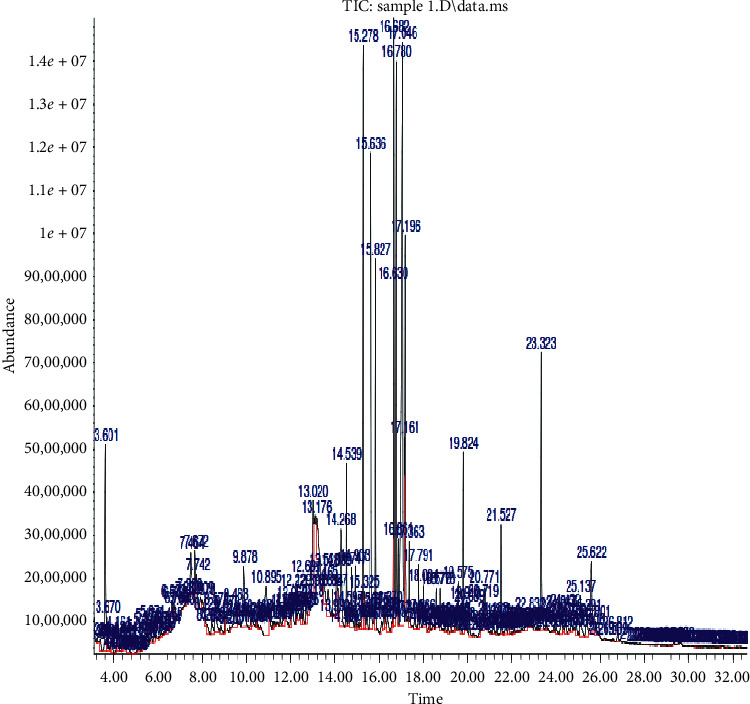
Mass spectrometry of the *Clerodendrum volubile* ethanol leaf extract (*CVE*) indicating molecular weights of each of the secondary metabolites and their relative abundance.

Figure 1 depicts the presence and relative abundance of thirty (30) different secondary metabolites in *CVE* obtained through gas chromatography-mass spectrometry, while the relatively abundant secondary metabolites present in *CVE* obtained through the phytoscan based on the CAS Library search include 9,12,15-octadecatrienoic acid (otherwise known as) (Z,Z,Z)-9,12,15-octadecatrienoic acid and (Z,Z,Z)-9,12,15-octadecatrienal (9.02%); urea, triethyl-urea (5.74%); 7-octadecenoic acid methyl ester and 9-octadecenoic acid methyl ester (5.61%); n-hexadecanoic acid (5.39%); ethyl-*α*-d-glucopyranoside, ethyl-*β*-d-glucopyranoside, and methyl-*β*-d-arabinopyranoside (5.13%); 9,12,15-octadecatrienoic acid and (Z,Z,Z)-ethyl- 9,12,15-octadecatrienoate (4.08%); phytol (3.28%); hexadecanoic acid, methyl ester (3.25%); methyl tetradecanoate (2.18%); glycine, N,N-dimethyl-, methyl ester and N,N-dimethyl-3-methoxypropylamine (2.16%); hexadecanoic acid, ethyl ester (2.03%); benzoic acid, 4-methoxy-benzoic acid, and 4-methoxy-benzoic acid 4-methoxy-(1.77%); 4-acetylanisole and 3-methoxyacetophenone (1.56%); 6-hydroxy-4,4,7*α*-trimethyl-5,6,7,7*α*-tetrahydrobenzofuran-2(4H)-one (1.36%); guaifenesin and 2-cyclohexen-1-one, 4-hydroxy-3,5, 6-trimethyl-4-(3-oxo-1-butenyl)-(1.32%); phthalic acid, di(2-propylpentyl) ester, phthalic acid, di(oct-3-yl) ester, and diisooctyl phthalate (1.19%); n-propyl 9,12,15-octadecatrienoate and 7,10,13-hexadecatrienoic acid (1.02%) (Table 3).

### 3.5. *In Vitro* Antioxidant Profiling of *CVE*

#### 3.5.1. Determination of DPPH-Scavenging Activity of *CVE*

Table 4 shows the *in vitro* DPPH-scavenging activities of 25 *μ*g/ml, 50 *μ*g/ml, 70 *μ*g/ml, and 100 *μ*g/ml of *CVE* in comparison with those of corresponding doses of the standard antioxidant drug (vitamin C) used. As shown, the DPPH-scavenging activities of the extract were significantly (*p* < 0.001) dose related and comparable to those of vitamin C.

#### 3.5.2. Determination of NO-Scavenging Activity of *CVE*

Table 5 shows the *in vitro* NO scavenging activities of 25 *μ*g/ml, 50 *μ*g/ml, 70 *μ*g/ml, and 100 *μ*g/ml of *CVE* in comparison with those of corresponding doses of the standard antioxidant drug (vitamin C). As shown, NO-scavenging activities of the extract were significantly (*p* < 0.001) dose related and comparable to those of vitamin C.

#### 3.5.3. Determination of FRAP of *CVE*

Table 6 shows the *in vitro* ferric-reducing activity power of 25 *μ*g/ml, 50 *μ*g/ml, 70 *μ*g/ml, and 100 *μ*g/ml of *CVE* in comparison with those of corresponding doses of the standard antioxidant drug. As shown, the FRAP activities of the extract were significantly (*p* < 0.001) dose dependent and comparable to those of the standard drug, vitamin C.

### 3.6. Effect of *CVE* on the Average Body Weight of DOX-Treated Rats

Table 7 shows the effect of repeated intraperitoneal DOX treatment and oral treatments with 50, 100, 200, and 400 mg/kg/day of *CVE* on the average body weight on day 1 and day 15 as well as percentage weight change (%∆wt.) of rats repeatedly treated with doxorubicin and *CVE* for 14 days. As shown in Table 7, repeated intraperitoneal DOX treatment was associated with significant and profound (*p* < 0.0001) reduction in the weight gain pattern in the DOX only treated (Group I) rats when compared to untreated control (normal) rats (Group VIII). With repeated oral treatment of 50, 100, and 200 mg/kg/day of *CVE*, there was a significant (*p* < 0.05 and *p* < 0.0001) dose-dependent attenuation in the weight loss pattern in the extract-treated rats (Table 7). Vitamin C also had a similar effect on the weight gain pattern in the DOX-treated rats (Table 7).

### 3.7. Effect of *CVE* on the Cardiac Tissue Oxidative Stress Markers (GSH, GST, GPx, SOD, CAT, and MDA) of DOX-Treated Rats

Repeated intraperitoneal injection of DOX to rats was associated with significant decreases (*p* < 0.05 and *p* < 0.0001) in the activities of SOD, CAT, GPx, GST, and GSH levels while significantly increasing (*p* < 0.001) MDA activities (Table 8). However, repeated oral treatment with *CVE* significantly (*p* < 0.05 and *p* < 0.0001) attenuated the alterations in the activities of these enzyme markers in the cardiac tissue (Table 8).

### 3.8. Effect of *CVE* on Cardiac Marker Enzymes (LDH and Troponin I) of DOX-Treated Rats

Repeated intraperitoneal injection of DOX resulted in significant increases (*p* < 0.05 and *p* < 0.0001) in the serum LDH and troponin I levels when compared to that of untreated negative (control) values (Table 9). However, oral pretreatments with 50–400 mg/kg/day of *CVE* significantly attenuated (*p* < 0.05 and *p* < 0.0001) increases in the serum LDH and troponin I levels (Table 9). Oral pretreatment with 20 mg/kg/day of vitamin C caused similar effects as *CVE* on the serum LDH and troponin I (Table 9).

### 3.9. Effect of *CVE* on the Serum Lipid (TG, TC, HDL-c, LDL-c, and VLDL-c) Level of DOX-Treated Rats

Repeated intraperitoneal DOX injections resulted in significant (*p* < 0.0001) increases in the serum TG and VLDL-c concentrations, while there were no significant alterations in the serum HDL-c and LDL-c concentrations in the treated rats when compared to those of untreated rats (Table 10). However, in rats repeatedly pretreated with 50–400 mg/kg/day of *CVE* orally, there were significant (*p* < 0.05 and *p* < 0.0001) dose-unrelated decreases in the serum concentration of TG and VLDL-c without significant (*p* > 0.05) alterations in the serum concentration of HDL-c and LDL-c when compared to DOX only treated rats (Table 10). It is also noteworthy that vitamin C had similar pattern of effects on the measured serum lipid parameters (Table 10).

### 3.10. Effect of *CVE* on Cardiovascular Risk Indices (AI and CRI) of DOX-Treated Rats

Repeated intraperitoneal injections with DOX resulted in a significant (*p* < 0.05) increase in the CRI value, while they had no significant effect on AI (Table 11). However, with pretreatment with 50–400 mg/kg/day of *CVE* orally, there were further significant (*p* < 0.05) dose-unrelated decreases in the CRI value without significant alterations in the AI values (Table 11). Oral pretreatments with 20 mg/kg/day of vitamin C had a similar pattern on the AI and CRI as exhibited by *CVE* (Table 11).

### 3.11. Histopathological Studies of the Effect of *CVE* on DOX-Treated Heart

**Figure 2:**
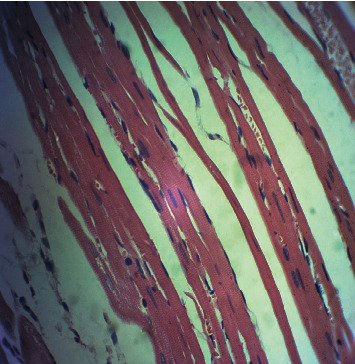
A cross-sectional representative of a DOX-intoxicated heart showing congested myocytes with scanty pyknotic and predominant hyperchromatic and meganuclei with interstitial fibrosis, suggestive of myocardial hypertrophy (×400 magnification, hematoxylin-eosin stain).

**Figure 3:**
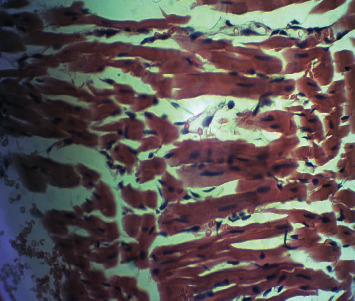
A cross-sectional representative of the normal rat heart showing normal cardiac architecture (×400 magnification, hematoxylin-eosin stain).

**Figure 4:**
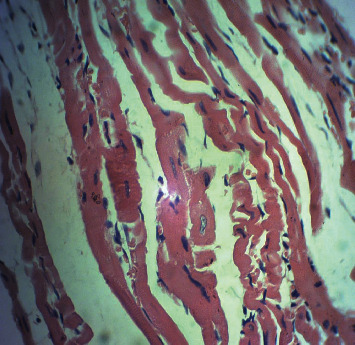
A cross-sectional representative of a DOX-intoxicated rat heart treated with 200 mg/kg/day *CVE* showing mildly congested myocytes with occasional meganuclei suggestive recovery from doxorubicin toxicity (×400 magnification, hematoxylin-eosin stain).

**Figure 5:**
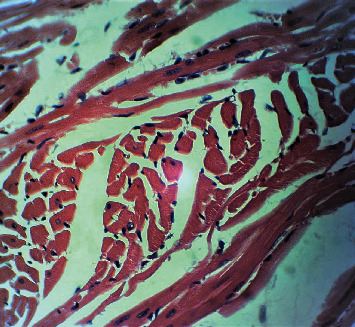
A photomicrograph of a cross-sectional representative of a DOX-intoxicated rat heart treated with 400 mg/kg/day *CVE* showing near normal myocytes with very scanty meganuclei indicating no remarkable histological changes (×400 magnification, hematoxylin-eosin stain).

**Figure 6:**
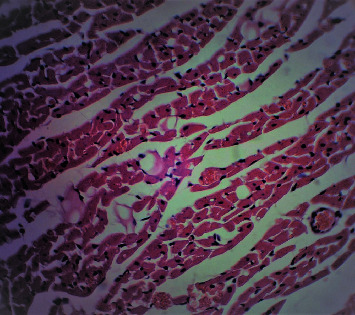
A photomicrograph of a cross-sectional representative of a doxorubicin-intoxicated rat heart treated with 20 mg/kg/day vitamin C showing mild congestion and scattered myocyte necrosis (×400 magnification, hematoxylin-eosin stain).

Figure 2 is a microphotograph of a cross-sectional representative of DOX only treated heart showing myocyte congestion with scanty pyknotic and predominant hyperchromatic and meganuclei with interstitial fibrosis, suggestive of myocardial hypertrophy when compared to normal untreated heart tissue with normal cardiac architecture (Figure 3). However, pretreatment with varying doses of *CVE* resulted in dose-related improvements in the histological distortions induced by DOX, especially at 200 mg/kg/day (Figure 4) and 400 mg/kg/day of *CVE* (Figure 5). On the contrary, there were histological features of mild congestion and scattered cardiac myocyte necrosis in rats pretreated with 20 mg/kg/day of vitamin C, suggesting persisting histological lesions induced by doxorubicin treatment even in the face of treatment with the standard antioxidant, vitamin C (Figure 6).

## 4. Discussion

DOX is a 14-hydroxylated congener of daunorubicin (the immediate biosynthetic DOX precursor) which acts by inhibiting the progression of topoisomerase II, an enzyme which relaxes supercoils in DNA for transcription, therefore inducing DNA double-strand breaks, especially in rapidly dividing cells, and resulting in DNA synthesis disruption [62]. DOX is known to accumulate majorly in the liver, the kidneys, and the heart [63]. However, the heart is highly susceptible to doxorubicin toxicity because of the high mitochondria-to-cardiomyocyte ratio, making it more susceptible to oxidative damage especially from semiquinone-type free radicals generated from DOX hepatic metabolism [63, 64]. In addition, the heart has a low regenerative capacity, compared to other body organs [63, 65]. These limiting factors make the heart susceptible to DOX-induced off-target cardiotoxicity, which is notoriously mediated by its highly reactive primary alcohol metabolite, doxorubicinol [66], leading to overwhelming oxidative stress and a compromised endogenous antioxidant system [67, 68]. The ensuing oxidative stress affects the lysosomes, microfibrils, mitochondria, and the sarcoplasmic reticulum [69], resulting in increased apoptotic cardiac myocytes and eventual cardiac cell damage [70]. The resulting cardiac damage is indicated by profound increases in the circulating specific and reliable cardiac biomarkers such as CK-MB, AST, Mb, IMA, PBNP, GPBB, troponin I, and LDH [71, 72]. Of these cardiac biomarkers, cardiotoxicity in this study was reliably measured using troponin I and LDH assays. Thus, the profound elevation in the serum activities of cardiac troponin I and LDH levels following doxorubicin treatment is a strong indication that DOX-induced cardiotoxicity was reliably established. The literature has it that doxorubicin induces its toxicity through generation of oxidative stress resulting in lipoperoxidation of the cardiac muscle with the ultimate leakage of troponin I and LDH into the serum [71–73]. Thus, our result is in complete agreement with reports of earlier studies by Al-Harthi et al. [73] and Ammar El-Sayed et al. [74]. The DOX-related biochemical changes were further corroborated by the histological features of congested myocytes with scanty pyknotic and predominant hyperchromatic and meganuclei with interstitial fibrosis, suggestive of myocardial hypertrophy. Again, this is in complete agreement with the report by Kwatra et al. [67]. However, profound attenuation in serum levels of troponin I and LDH following oral pretreatment with 50–400 mg/kg/day of *CVE* is a clear demonstration of the protective potential of the extract against DOX-associated cardiotoxicity. Similarly, attenuations in the serum troponin I and LDH were also corroborated by the significant improvement in the histological architecture of the *CVE*-treated heart muscle especially at the oral dose of 400 mg/kg/day of *CVE* which showed no remarkable histological lesions in the *CVE*-treated heart.

Another notable finding in this current study is that the increased activities of the oxidative markers induced following doxorubicin treatment evident from marked increases in lipid peroxidation products (MDA) depleted reduced GSH levels and decreased SOD and CAT activities. GSH is considered to be the most important intracellular antioxidant system that is utilized for the inactivation of lipid peroxides through the activity of GPx, which generates GSSG as a byproduct [75]. It also plays a vital role in conjugating GST, which detoxifies the reactive substances of lipid peroxidation and other xenobiotics [76]. Therefore, GSH depletion leads to loss of cellular integrity and damage to macromolecules such as membrane lipids and also accumulation of its oxidized form (GSSG) which further leads to electrical and contractile dysfunction. DOX and its metabolites cause depletion of the cardiac GSH level, resulting in the consistent formation of oxygen-free radicals [77]. Similarly, SOD and CAT play a critical role in combating DOX-induced oxidative stress. Superoxide radicals generated at the site of damage can alter the SOD and CAT levels which may predispose to accumulation of the superoxide anion and in turn damage the myocardium. In the present study, it is suggested that DOX-induced massive free radical production which utilized the myocardium oxidative machinery that further leads to reduction in the activities of antioxidant enzymes via inhibition of enzyme protein biosynthesis. These results are consistent with those previously reported by Li et al. [78] and Mantawy et al. [79]. The fact that *CVE* treatment could significantly reduce the DOX-induced lipid peroxidation, raise the reduced GSH level, and increase the activities of SOD and CAT strongly indicates the profound antioxidant property of the extract which was further corroborated by the results of the *in vitro* antioxidant study. Already, the antioxidant and free radical-scavenging activities of *CVE* are well documented [32, 80–82] Again, the literature has shown polyphenols (consisting of flavonoids, alkaloids, tannins, and anthocyanins) to freely scavenge free radicals in the body [83–86]. Among the secondary metabolites identified, the most prevailing compounds which have been reported for eliciting potent antioxidant activities for other plant extracts were tetradecanoic acid [87], n-hexadecanoic acid and hexadecanoic acid methyl and ethyl esters [88], phytol [89], and gamma-tocopherol [90]. These antioxidant compounds are known to possess redox properties through their free radical adsorption and neutralization [91, 92]. However, other reported pharmacological activities attributable to the identified secondary metabolites include anti-inflammatory, antimicrobial, cytotoxic, antihyperglycemic, hypocholesterolemic, and antiapoptotic activities [93, 94]. Thus, the presence of these identified secondary metabolites in high amount as indicated by the results of quantitative analysis of *CVE* undertaken in this study could be responsible for the prominent antioxidant/free radical scavenging and hypocholesterolemic activities, as well as profound improvement in the cardiovascular disease risk indices recorded in this study.

Other findings of significance are the profound increases in the serum TG, TC, and VLDL-c induced by DOX treatment. These findings are in tandem with those previously reported where prolonged treatment of experimental rats with DOX resulted in significant increases in the serum lipids, especially cholesterol and triglycerides [95–97]. The molecular mechanism of these lipid derangements has been reported to be mediated via downregulation of PPAR*γ*, mainly the white adipose tissue receptor, which regulates the expression of glucose and fatty acid transporters and plays a crucial role in lipid storage and glucose metabolism [98]. Thus, the downregulation of PPAR*γ* inhibits blood glucose and lipid clearance, thereby causing hyperglycemia and hyperlipidemia [99]. Research studies have equally shown that derangements in the lipid profile predispose to cardiovascular diseases and increase the risk of major cardiovascular diseases such as ischemic heart disease and thrombotic stroke [100–103] which was corroborated in the present study by the profound increased CRI value, which itself is a reliable indicator of cardiovascular diseases, especially ischemic heart diseases. Drugs have also been reported to cause dyslipidemia-related heart diseases including doxorubicin [103, 104]. It is equally well documented in the literature that a direct relationship exists between hypercholesterolemia and hyperlipidemia and atherosclerosis which is considered as a major cause of cardiovascular disease, especially coronary heart disease [105–107]. Thus, drugs including medicinal plants with hypolipidemic/antihyperlipidemic activities could equally be considered antiatherosclerotic and cardioprotective [108–110]. Thus, the hypolipidemic/antihyperlipidemic activity of *CVE* observed in this study is in tandem with those previously reported for *Clerodendrum volubile* and related species [32, 80, 111–114]. The fact that *CVE* profoundly lowered the serum TG, VLDL-c, and CRI is a further demonstration of the cardioprotective potential of *CVE* against DOX-induced cardiotoxicity and DOX-induced dyslipidemia which is similar to an earlier report by Kulkarni and Viswanatha-Swamy [115]. Thus, it is plausible for *CVE* to be lowering serum lipids and improving CRI value either by promoting its clearance from the body or inhibiting its *de novo* biosynthesis. Again, the serum lipid-lowering effect of *CVE* is attributable to its high polyphenolic content which has been widely reported to attenuate dyslipidemia in different experimental models [79, 116, 117].

## 5. Conclusions

Overall, it can be safely concluded that *CVE* offers protection in DOX-induced cardiotoxicity that was mediated via free radical-scavenging activity/antioxidant mechanism and improvements in the cardiovascular disease risk indices.

## Figures and Tables

**Table 1:** Group treatment of rats.

Groups	Treatments
Group I	10 ml/kg of distilled water *p.o.* for 14 days + 2.5 mg/kg of doxorubicin hydrochloride in 0.9% normal saline given *i.p.* on alternate days for 14 days
Group II	200 mg/kg/day of *Clerodendrum volubile* ethanol leaf extract in 5% DMSO-distilled water *p.o.* for 14 days + 1 ml/kg of 0.9% normal saline given *i.p.* on alternate days for 14 days
Group III	50 mg/kg/day of *Clerodendrum volubile* ethanol leaf extract in 5% DMSO-distilled water *p.o.* for 14 days + 2.5 mg/kg of doxorubicin hydrochloride in 0.9% normal saline given *i.p.* on alternate days for 14 days
Group IV	100 mg/kg/day of *Clerodendrum volubile* ethanol leaf extract in 5% DMSO-distilled water *p.o.* for 14 days + 2.5 mg/kg of doxorubicin hydrochloride in 0.9% normal saline given *i.p.* on alternate days for 14 days
Group V	200 mg/kg/day of *Clerodendrum volubile* ethanol leaf extract in 5% DMSO-distilled water *p.o.* for 14 days + 2.5 mg/kg of doxorubicin hydrochloride in 0.9% normal saline given *i.p.* on alternate days for 14 days
Group VI	400 mg/kg/day of *Clerodendrum volubile* ethanol leaf extract in 5% DMSO-distilled water *p.o.* for 14 days + 2.5 mg/kg of doxorubicin hydrochloride in 0.9% normal saline given *i.p.* on alternate days for 14 days
Group VII	20 mg/kg/day of vitamin C in distilled water *p.o.* for 14 days + 2.5 mg/kg of doxorubicin hydrochloride in 0.9% normal saline given *i.p.* on alternate days for 14 days
Group VIII	10 ml/kg/day of distilled water *p.o.* for 14 days + 1 ml/kg of 0.9% normal saline given *i.p*

**Table 2:** Quantitative analysis of the secondary metabolites in *CVE* (mg/100 mg of the dry extract sample).

Secondary metabolite	Quantity (mg/100 mg of dry extract)
Flavonoids	34.79 ± 0.37
Alkaloids	36.73 ± 0.27
Reducing sugars	07.78 ± 0.09
Cardiac glycosides	24.55 ± 0.12

**Table 3:** Quantitative analysis of the secondary metabolites (PhytoScan) of the *Clerodendrum volubile* ethanol leaf extract (*CVE*) using gas chromatography-mass spectrometry.

Pk#	RT	Area (%)	Library/ID ref#	CAS#	Quality
(1)	3.601	2.16	Glycine, N,N-dimethyl-, methyl ester	8679 007148-06-3	86
			N,N-Dimethyl-3-methoxypropylamine	8722 020650-07-1	80
			2-Methyl-allyl ethyl ether	1759 000557-31-3	50
			Dimethylaminomethyl-isopropyl-sulfide	14996 077422-33-4	50
(2)	7.464	5.74	Urea, triethyl-urea	21278 019006-59-8	50
(3)	8.579	0.77	5-Hydroxymethylfurfural	11338 000067-47-0	93
			5-Hydroxymethylfurfural	11339 000067-47-0	70
			5-Hydroxymethylfurfural	11337 000067-47-0	62
(4)	9.878	1.56	4-Acetylanisole	25100 000100-06-1	94
			3-Methoxyacetophenone	25121 000586-37-8	76
(5)	10.895	1.77	Benzoic acid	26636 000100-09-4	96
			4-Methoxy-benzoic acid	26633 000100-09-4	95
			4-Methoxy-benzoic acid 4-methoxy	26632 000100-09-4	93
(6)	12.691	0.50	Megastigmatrienone	56052 038818-55-2	99
			1H-1,5-Benzodiazepine, 2,3,4,5-tetrahydro-2,2,4-trimethyl-phenol	56033 040358-38-1	70
			2-(1,1-Dimethyl-2-propenyl)-3,6-dimethyl-	56097 092617-73-7	62
(7)	13.113	5.13	Ethyl-*α*-d-glucopyranoside	72939 019467-01-7	74
			Ethyl-*β*-d-glucopyranoside	60076 000709-50-2	53
			Methyl-*β*-d-arabinopyranoside	35240 005328-63-2	50
(8)	13.528	2.18	Methyl tetradecanoate	104286 000124-10-7	74
(9)	13.684	0.95	Germacyclopentane	36709 004554-75-0	53
(10)	13.887	0.81	Tetradecanoic acid	91415 000544-63-8	97
(11)	14.089	1.36	6-Hydroxy-4,4,7*α*-trimethyl-5,6,7,7 *α*-tetrahydrobenzofuran-2(4H)-one	61438 073410-02-3	93
(12)	14.268	1.32	Guaifenesin	62883 000093-14-1	72
			2-Cyclohexen-1-one, 4-hydroxy-3,5,6-trimethyl-4-(3-oxo-1-butenyl)	85356 077846-84-5	64
(13)	14.539	0.88	Neophytadiene	138502 000504-96-1	99
			Bicyclo[3.1.1]heptane	17424 006876-13-7	55
			2,6,6-Trimethyl-, (1.alpha.,2.beta.,5.alpha.)9-octadecyne	111836 035365-59-4	53
(14)	14.747	0.67	9-Octadecen-1-ol	128820 000143-28-2	70
			(Z)-6-Octen-1-ol, 3,7-dimethyl	51056 000105-85-1	70
			Formate-6-octen-1-ol,3,7-dimethyl-, formate	51061 000105-85-1	70
(15)	15.278	3.25	Hexadecanoic acid, methyl ester	130821 000112-39-0	98
(16)	15.636	5.39	n-Hexadecanoic acid	117419 000057-10-3	99
(17)	15.827	2.03	Hexadecanoic acid, ethyl ester	144309 000628-97-7	99
(18)	16.682	5.61	7-Octadecenoic acid, methyl ester	155720 057396-98-2	99
			9-Octadecenoic acid, methyl ester	155758 001937-62-8	99
			(E)-9-Octadecenoic acid, methyl ester	155754 001937-62-8	99
(19)	16.780	3.28	Phytol	155849 000150-86-7	98
(20)	17.046	9.02	9,12,15-Octadecatrienoic acid	138418 000463-40-1	99
			(Z,Z,Z)-9,12,15-Octadecatrienoic acid	138420 000463-40-1	99
			(Z,Z,Z)-9,12,15-Octadecatrienal	123143 026537-71-3	91
(21)	17.196	4.08	9,12,15-Octadecatrienoic acid	165643 001191-41-9	99
			(Z,Z,Z)-Ethyl 9,12,15-octadecatrienoate	165627 1000336-77-4	99
			9,12,15-Octadecatrienoic acid	165642 001191-41-9	99
(23)	18.004	0.62	Ethyl-9-hexadecenoate	142080 054546-22-4	89
			Cyclopentadecanone, 2-hydroxy-	102369 004727-18-8	70
(24)	19.575	0.54	Hexadecanoic acid	188252 023470-00-0	90
			2-Hydroxy-1-(hydroxymethyl)ethyl ester	188251 023470-00-0	87
(25)	19.824	1.19	Phthalic acid, di(2-propylpentyl)ester	233419 1000377-93-5	91
			Phthalic acid, di(oct-3-yl) ester	233383 1000377-72-3	80
			Diisooctyl phthalate	233361 000131-20-4	74
(26)	20.771	1.02	n-Propyl 9,12,15-octadecatrienoate	179097 1000336-79-4	93
			7,10,13-Hexadecatrienoic acid, methyl ester	124916 056554-30-4	91
			Methyl (Z)-5,11,14,17-eicosatetraenoate	177257 059149-01-8	90
(27)	21.527	0.68	Squalene	243222 000111-02-4	99
(28)	22.832	0.55	Gamma-tocopherol	245804 007616-22-0	95
(29)	25.137	0.68	2-(4-Fluoro-phenyl)-4-(3-methyl-benzylidene)-4H-oxazol-5-one	140918 1000296-71-2	55
(30)	25.801	0.52	4-Dehydroxy-N-(4,5-methylene dioxy-nitrobenzylidene) tyramine	157264 1000111-66-9	53

Pk#: peak number, RT: retention time, area%: percentage area covered, library/ID ref#: library/identification number, and CAS#: chemical abstract scheme number.

**Table 4:** *In vitro* DPPH-scavenging activity of 25–100 *μ*g/ml of *CVE* and vitamin C.

Drug	Graded doses (*μ*g/ml)			
	25	50	75	100
*CVE*	33.41 ± 0.42	47.10 ± 0.63^c^	60.69 ± 0.52^c^	76.25 ± 0.32^c^
Vit. C	45.05 ± 0.48	56.55 ± 0.96^c^	70.45 ± 0.48^c^	89.83 ± 0.36^c^

^c^A significant increase at *p* < 0.001.

**Table 5:** *In vitro* nitric oxide- (NO-) scavenging activity of 25–100 *μ*g/ml of *CVE* and vitamin C.

Drug	Graded doses (*μ*g/ml)			
	25	50	75	100
*CVE*	20.52 ± 0.34	49.32 ± 0.57^c^	64.97 ± 0.34^c^	78.57 ± 0.57^c^
Vit. C	47.38 ± 0.26	62.61 ± 0.10^c^	71.57 ± 1.32^c^	84.91 ± 0.53^c^

^c^A significant increase at *p* < 0.001.

**Table 6:** *In vitro* FRAP activities of 25–100 *μ*g/ml of *CVE* and vitamin C.

Drug	Graded doses (*μ*g/ml)			
	25	50	75	100
*CVE*	0.13 ± 0.00	0.24 ± 0.00^c^	0.33 ± 0.00^c^	0.41 ± 0.00
Vit. C	0.18 ± 0.00	0.40 ± 0.00^c^	0.51 ± 0.00^c^	0.66 ± 0.00

^c^A significant increase at *p* < 0.001.

**Table 7:** Effect of repeated oral treatment with 50–400 mg/kg/day of *CVE* on the average body weight of DOX-treated rats.

Group	Body wt. on day 1 (g)	Body wt. on day 14	% ∆wt.
I	159.9 ± 15.1	143.1 ± 19.0	−10.7 ± 04.7^c−^
II	178.3 ± 18.0	191.1 ± 20.6	07.1 ± 04.1^c+^
III	144.2 ± 15.8	141.5 ± 25.2	−02.4 ± 10.5^a+^
IV	163.9 ± 08.0	153.0 ± 11.0	−06.6 ± 04.8^a+^
V	154.0 ± 19.6	151.6 ± 23.1	09.0 ± 05.5^c+^
VI	165.2 ± 12.4	147.9 ± 12.8	−10.6 ± 05.8
VII	151.3 ± 12.9	146.0 ± 15.4	−03.5 ± 04.5^a+^
VIII	163.8 ± 16.8	187.4 ± 20.2	14.5 ± 06.2

^c−^A significant decrease at *p* < 0.0001 when compared to the untreated (normal) negative control (Group VIII). ^c+^A significant increase at *p* < 0.0001 when compared to the untreated (DOX-treated) negative control (Group I). ^a+^A significant increase at *p* < 0.05 when compared to the untreated (DOX-treated only) negative control (Group I).

**Table 8:** Antioxidant activities of 50–400 mg/kg/day of *CVE* in DOX-treated rat cardiac tissue.

Groups	Antioxidant parameters					
	GSH	GST	GPx	SOD	CAT	MDA
I	88.1 ± 5.3^c−^	20.4 ± 0.1^c−^	81.7 ± 4.9^c−^	2.0 ± 0.2^a−^	13.3 ± 1.7^a−^	2.8 ± 0.1
II	62.8 ± 4.9^f−^	21.4 ± 0.9^f−^	69.4 ± 5.4	2.2 ± 0.1	11.3 ± 0.5	2.1 ± 0.3
III	51.2 ± 2.8^c−^	20.8 ± 0.3^f−^	51.5 ± 3.1^f−^	3.3 ± 0.2	17.9 ± 1.1	3.5 ± 0.4
IV	77.6 ± 5.7	30.8 ± 0.7^c+^	81.4 ± 5.4	5.5 ± 1.1^c+^	35.6 ± 4.1^c+^	4.3 ± 0.3^b+^
V	45.6 ± 4.2^c−^	21.5 ± 0.4^f−^	50.3 ± 4.6^f−^	4.0 ± 0.2^a+^	21.8 ± 1.6^a+^	3.9 ± 0.2^a+^
VI	65.7 ± 4.3^c−^	22.3 ± 0.8^f−^	64.6 ± 4.0^d−^	2.8 ± 0.3	19.6 ± 0.7	6.4 ± 0.7^c+^
VII	112.6 ± 2.3^c+^	21.9 ± 0.3^f−^	111.1 ± 2.3^c+^	5.3 ± 0.2^c+^	40.2 ± 1.7^c+^	4.5 ± 0.5^b+^
VIII	113.5 ± 2.2	23.6 ± 0.4	111.9 ± 2.2	2.5 ± 0.2	19.8 ± 1.5	2.4 ± 0.2

^a−,^ ^c−^Significant decreases at *p* < 0.05 and *p* < 0.0001, respectively, when compared to the untreated negative (normal) control value. ^c+^A significant increase at *p* < 0.0001 when compared to the untreated negative (normal) control values. ^a+,b+^Significant increases at *p* < 0.05 and *p* < 0.001, respectively, when compared to the untreated negative (normal) control values. ^d−,^^f−^Significant decreases at *p* < 0.05 and *p* < 0.0001 when compared to the untreated positive (doxorubicin treated only) control values, respectively.

**Table 9:** Effect of 50–400 mg/kg/day of *CVE* on serum LDH and cardiac troponin I in DOX-intoxicated rats.

Treatment groups	LDH (U/L)	Troponin I (ng/ml)
I	4204 ± 637.1^a+^	21.18 ± 7.72^c+^
II	3734 ± 251.5	3.29 ± 2.41^f–^
III	2781 ± 657.5^d–^	7.93 ± 7.03^d–^
IV	2939 ± 184.1^d–^	10.63 ± 6.7^d–^
V	2530 ± 189.2^d–^	2.96 ± 1.71^f–^
VI	2214 ± 340.1^d–^	3.53 ± 1.84^f–^
VII	2907 ± 204.4^d–^	3.46 ± 1.71^f–^
VIII	3331 ± 227.5	4.11 ± 2.59

^a+,^ ^c+^Significant increases at *p* < 0.05 and *p* < 0.0001, respectively, when compared to the untreated negative (normal) control values. ^d−,^^f−^Significant decreases at *p* < 0.05 and *p* < 0.0001, respectively, when compared to the untreated positive (doxorubicin treated only) control values, respectively.

**Table 10:** Effect of 50–400 mg/kg/day of *CVE* on the serum lipid profile of DOX-treated rats.

Groups	Serum lipids				
	TG (mmol/l)	TC (mmol/l)	HDL-c (mmol/l)	LDL-c (mmol/l)	VLDC-c (mmol/l)
I	1.3 ± 0.1^c+^	2.3 ± 0.1^a+^	0.6 ± 0.0	1.2 ± 0.1	1.3 ± 0.1^c+^
II	0.8 ± 0.2^d−^	1.8 ± 0.2^d−^	0.6 ± 0.0	0.9 ± 0.1^d−^	0.4 ± 0.1^f−^
III	0.8 ± 0.1^d−^	2.3 ± 0.1	0.7 ± 0.0	1.3 ± 0.1	0.5 ± 0.1^f−^
IV	1.1 ± 0.1	2.3 ± 0.1	0.6 ± 0.0	1.2 ± 0.1	0.5 ± 0.1^f−^
V	0.8 ± 0.1^d−^	2.3 ± 0.1	0.6 ± 0.0	1.3 ± 0.1	0.4 ± 0.0^f−^
VI	1.0 ± 0.1^d−^	2.3 ± 0.2	0.6 ± 0.0	1.2 ± 0.1	0.5 ± 0.1^f−^
VII	0.9 ± 0.1^d−^	2.1 ± 0.1	0.6 ± 0.0	1.1 ± 0.1	0.4 ± 0.0^f−^
VIII	0.6 ± 0.1	2.0 ± 0.1	0.6 ± 0.0	1.1 ± 0.0	0.3 ± 0.0

^a+,^ ^c+^Significant increases at *p* < 0.5 and *p* < 0.0001, respectively, when compared to the untreated negative (normal) control value. ^d−,^^f−^Significant decreases at *p* < 0.05 and *p* < 0.0001 when compared to the untreated positive (doxorubicin treated only) control values, respectively.

**Table 11:** Effect of 50–400 mg/kg/day of *CVE* on the atherogenic index (AI) and the coronary artery index (CRI) in DOX-intoxicated rats.

Treatment groups	AI	CRI
I	2.1 ± 0.1	4.0 ± 0.2^a+^
II	1.6 ± 0.2	3.2 ± 0.2^e−^
III	2.0 ± 0.2	3.5 ± 0.2^d−^
IV	1.9 ± 0.3	3.9 ± 0.3
V	2.1 ± 0.1	3.8 ± 0.1^d−^
VI	2.0 ± 0.1	3.8 ± 0.2^d−^
VII	1.8 ± 0.1	3.8 ± 0.2^d−^
VIII	1.1 ± 0.0	3.5 ± 0.1

^a+^A significant increase at *p* < 0.05 when compared to the untreated negative (normal) control values. ^d−,^^e−^Significant decreases at *p* < 0.05 and *p* < 0.01, respectively, when compared to the untreated positive (doxorubicin treated only) control values, respectively.

## Data Availability

The data used to support the study can be available upon request to the corresponding author.

## Abbreviations

AI: Atherogenic index
AST: Aspartate transaminase
CAT: Catalase
CK-MB: Creatine kinase
CRI: Coronary artery index
*CVE*: *Clerodendrum volubile* ethanol leaf extract
DMSO: Dimethyl sulfoxide
DOX: Doxorubicin
DPPH: 1,1-Diphenyl-2-picrylhydrazyl
DTNB: 5,5-Dithiobisnitro benzoic acid
GPBB: Glycogen phosphorylase isoenzyme BB
GPx: Glutathione peroxidase
GSH: Reduced glutathione
GST: Glutathione S-transferase
FRAP: Ferric-reducing activity power
HCl: Hydrochloric acid
HDL-c: High-density lipoprotein cholesterol
IMA: Ischemic modified albumin
*i.p.*: Intraperitoneal
KCl: Potassium chloride
LDH: Lactate dehydrogenase
LDL-c: Low-density lipoprotein cholesterol
MAD: Malondialdehyde
Mb: Myoglobin
NO: Nitric oxide
PBNP: Probrain natriuretic peptide
*p.o.*: *Per os*
PPAR*γ*: Peroxisome proliferator-activator receptor gamma
SEM: Standard error of the mean
SOD: Superoxidase dismutase
TBA: Thiobarbituric acid
TC: Total cholesterol
TCA: Tricarboxylic acid
TG: Triglyceride
UNILORIN: University of Ilorin
UV: Ultraviolet
Vit. C: Vitamin C
VLDL-c: Very low-density lipoprotein cholesterol.
